# Cysteine Cathepsins in Tumor-Associated Immune Cells

**DOI:** 10.3389/fimmu.2019.02037

**Published:** 2019-08-28

**Authors:** Tanja Jakoš, Anja Pišlar, Anahid Jewett, Janko Kos

**Affiliations:** ^1^Faculty of Pharmacy, University of Ljubljana, Ljubljana, Slovenia; ^2^UCLA School of Dentistry and Medicine, Los Angeles, CA, United States; ^3^Department of Biotechnology, Jožef Stefan Institute, Ljubljana, Slovenia

**Keywords:** cysteine cathepsins, cystatins, immune response, tumor-associated macrophages (TAM), myeloid-derived suppressor cells (MDSC)

## Abstract

Cysteine cathepsins are key regulators of the innate and adaptive arms of the immune system. Their expression, activity, and subcellular localization are associated with the distinct development and differentiation stages of immune cells. They promote the activation of innate myeloid immune cells since they contribute to toll-like receptor signaling and to cytokine secretion. Furthermore, they control lysosomal biogenesis and autophagic flux, thus affecting innate immune cell survival and polarization. They also regulate bidirectional communication between the cell exterior and the cytoskeleton, thus influencing cell interactions, morphology, and motility. Importantly, cysteine cathepsins contribute to the priming of adaptive immune cells by controlling antigen presentation and are involved in cytotoxic granule mediated killing in cytotoxic T lymphocytes and natural killer cells. Cathepins'aberrant activity can be prevented by their endogenous inhibitors, cystatins. However, dysregulated proteolysis contributes significantly to tumor progression also by modulation of the antitumor immune response. Especially tumor-associated myeloid cells, such as tumor-associated macrophages and myeloid-derived suppressor cells, which are known for their tumor promoting and immunosuppressive functions, constitute the major source of excessive cysteine cathepsin activity in cancer. Since they are enriched in the tumor microenvironment, cysteine cathepsins represent exciting targets for development of new diagnostic and therapeutic moieties.

## Introduction

Lysosomes are specialized recycling organelles responsible for the breakdown of material that enters endosomes or autophagosomes. They contain more than 50 different hydrolytic enzymes. The lysosomal lumen has a slightly acidic pH of around 4–5 that is required for optimal activity of lysosomal peptidases. The main group of lysosomal peptidases, the so-called cathepsins, comprises 2 serine peptidases (cathepsins A and G), 2 aspartic peptidases (cathepsins D and E), and 11 members of the cysteine peptidases (cathepsins B, C, F, H, K, L, O, S, V, W, and X). The latter have received a lot of attention, as they also play a role outside the lysosomes. They are involved in numerous non-redundant tissue and cell type specific physiological processes located in the nucleus, the cell cytosol, at the cell membrane or in the extracellular space (1–3). Proteolysis is used by cells for regulating protein functions and needs to be under strict control (4). Otherwise, excessive activity, mislocalization, or changed expression of cysteine cathepsins can lead to severe pathological conditions. Most notably, cysteine cathepsins can account for the progression of cardiovascular, neurodegenerative diseases, autoimmune disorders, and cancer (5, 6). Unresolved inflammation is a common hallmark of such disorders and targeting the immune system is seen as a promising therapeutic strategy. One of the most prominent roles of cysteine cathepsins is the regulation of key functions of the immune system. Imbalance of cysteine cathepsins' activity can lead to impaired immunity resulting, detrimentally, in augmented or attenuated immune responses, as in the case of autoimmune disorders and cancer, respectively. The following review delineates the role of cysteine cathepsins in regulating the various aspects of immune cell function in physiological conditions and during cancer.

## Cysteine cathepsins

### Structure

Cysteine cathepsins possess, typically, a two-domain papain fold structure and act as endopeptidases, with the exception of exopeptidases cathepsins X and C. In addition, a dual exo/endopepeptidase activity is attributable to cathepsins B and H (2). Distinct structural elements are required in exopeptidases in order to obstruct the active-site cleft, thereby preventing the binding of extended substrates. For example, a mini loop and a pH sensitive occluding loop confer carboxypeptidase activities to cathepsins X and B, respectively. On the other hand, the aminopeptidases cathepsins H and C utilize parts of their pro-regions to dock the N-terminus of the substrate to the active site cleft (1, 7).

### Localization

Some of the cysteine cathepsins such as cathepsins B, C (also known as cathepsin O2 or dipeptidyl peptidase I), F, H, L, and O are expressed ubiquitously in the human body, whereas others are restricted to specific cells and tissues. For example, cathepsin K is expressed highly in osteoclasts and, to a smaller degree, in macrophages, some fibroblasts and epithelial cells (8). Cathepsin W (lymphopain) is found exclusively in cytotoxic cells, at higher levels in natural killer (NK) cells and, to a lesser extent, in CD8+ T lymphocytes (9). Expression of cathepsin X (also known as cathepsin Z or cathepsin P) is restricted predominantly to the immune cells, especially to those of the myeloid lineage (10). The same holds for cathepsin S which is typically present in antigen presenting cells (APC) such as dendritic cells (DC) and B lymphocytes (11). Cathepsin V (also termed cathepsin L2) is highly homologous to cathepsin L but is expressed only in thymus and testis (12).

### Biological Functions

#### In the Extracellular Milieu

Even though cysteine cathepsins show broad and redundant substrate specificities, they can carry out highly specialized functions. This can be achieved through strict regulation of their expression and activity in distinct subcellular compartments. It has been shown that, in cytoplasm, nucleus or extracellular environment and, in spite of suboptimal pH conditions, they still retain their proteolytic activities. Glycosaminoglycans are known to accelerate the autocatalytic activation of cathepsins and to improve their stability in the extracellular milieu (13). Further, the activity of secreted cathepsins can be enhanced in the acidic tumor microenvironment and in bone lacuna (14). Cathepsins can circumvent endo-lysosomal sorting, employing alternative ways of cell trafficking. At least for cathepsins B and L, it has been demonstrated that they can be secreted without entering the endo/lysosomal pathway (15). Secreted and membrane bound cathepsins, in particular cathepsins B, S, L and K, may contribute to degradation of the extracellular matrix (ECM), either directly or as initiators of the proteolytic cascade (16). In this way they can contribute to processes like wound healing, bone remodeling, immune cell infiltration, maintenance of stem cell niches, or tumor invasion and migration (14, 16, 17). In tumor cells, intracellular degradation of ECM by cysteine cathepsins has also been shown to contribute additionally to their invasive potential (18). Furthermore, extracellular cathepsins may alter cell adhesive and migratory properties by modulating integrin receptor interactions with ECM (19). Cathepsins can also modulate cell trafficking and recruitment through cleavage of chemokines (20). They can influence signaling pathways by shedding ectodomains of transmembrane receptors (21) and by releasing growth factors (22, 23). The role of cysteine cathepsins is summarized in Table 1.

**Table 1:** The role of cysteine cathepsins in immune cells.

**Myeloid cells**	
Antigen processing and antigen presentation	Cathepsin S processes antigens for TAP-independent cross-presentation on MHC I (24). Redundant cathepsins are involved in generating peptides for MHC II presentation (25). Cathepsin L contributes importantly to the peptide repertoire presented by MHC II in cortical thymic epithelial cells (26). Cathepsins L and S play a non-redundant role in cleaving Ii in cortical epithelial cells and DC, macrophages or B cells, respectively (27).
Autophagy and survival	Cathepsin B cleaves the calcium flux channel (TRPML1) and regulates the activity of transcription factor TFEB, thereby influencing lysosomal biogenesis (28). Autophagy and cysteine cathepsins support the survival of differentiating monocytes (29). Autophagy and cathepsin S activity maintain macrophages in the M2 polarized state (30).
TLR activation and cytokine secretion	Several cathepsins (B, L, H, S, and F) are needed for the activation of TLR-3,−7 and−9 (31–33). Cathepsin K contributes to maximal secretion of cytokine IL-6 and cathepsin L enhances non-oxidative killing of *Staphylococcus aureus* by infected macrophages (34). Cathepsin B regulates IL-12 secretion from DC and from macrophages in *Leishmania major* infected mice (35). Cathepsin B controls the production of IL-1β and IL-18 by activating NLRP3 inflammasome (36, 37). Cathepsin B controls the trafficking of TNF-α vesicles and the secretion of TNF-α in monocytes (38).
Migration and adhesion	Endopeptidases (cathepsins B, S, L, K) cleave ECM components, thus facilitating immune cell migration (14). Pro- and mature forms of cathepsin X interact with β_2_ integrin receptors, thus modulating their affinity for extracellular ligands (19). Mature cathepsin X cleaves sequentially four C-terminal amino acids from the β2 integrin cytoplasmic tail, thus regulating the adhesion and phagocytosis of U937 macrophages (39) as well as the adhesion of maturing DC via Mac-1 (40). Cathepsins B, L, S, and K activate angiogenic and neutrophils attracting ELR-CXC chemokines ad inactivate angiostatic and T lymphocyte attracting non-ELR chemokines (20). Cathepsin X cleaves CXCL12 and helps hematopoietic stem cells detachment from osteoclasts (41).
TAM	Generally elevated cathepsins' activity was observed in TAM. Cathepsin K modulates the immune response in a SCID-hu mouse bone tumor model by recruiting TAM via CCL2 and promoting expression of cathepsin B and COX2 (42). Several cathepsins can process COX2 in its mature form, thereby influencing immunosupressive PGE2 production (43). Cathepsin S releases CD74 intracellular domain, which in turn activates NF-kB-dependent CCL2 transciption (44). Cathepsins B and S, secreted from TAM, protect tumor cells from paclitaxel, etoposide and doxorubicin induced apoptosis (45).
MDSC	Cathepsin B has been shown to be important for the generation of MDSC in a mouse model of hereditary polyposis (46). Cathepsins B, L, and X prevent the fusion of MDSC-derived osteoclast and therefore reduce their osteolytic activity (47). Cathepsin B reduces the effectiveness of the chemotherapeutic drugs 5-fluorouracil and gemcitabine by activating NLRP3 inflammasome-dependent IL-17 production (48). Reduced levels of cathepsin B inhibitor NGP in MDSC from metastatic mouse tumors have been reported (49).
**T lymphocytes, NK cells**	
Migration, morphology, and immunological synapse	Cathepsin X modulates the activity of integrin LFA-1, enabling lymphocytes to form elongated extensions, nanotubes (50), thus enhancing lymphocyte migration (51). Cathepsin X is important for directing the immune response by modulating the Mac-1 receptor on macrophages or DC and LFA-1 on T lymphocytes (52). Cathepsin X influences the binding of the adaptor molecules talin 1 (53) and α-actinin (54) to the cytoplasmic domain of LFA-1 and regulates profilin 1 (55) binding to the actin cytoskeleton, which could be important in stabilizing the immunological synapse during antigen presentation or target cell lysis (56, 57).
Cytotoxicity	Cathepsins L (58) and C (59) activate pro-perforin and cathepsins C (60) and H (61) activate pro-granzymes A and B in cytotoxic granules of CTL and NK cells. Cathepsin L cleaves complement component C3, generating C3a and C3b fragments that signal, in an autocrine manner, CD4+ lymphocyte proliferation and Th1 differentiation (62) as well as induce maximal cytotoxic activity of CD8+ lymphocytes (63). Cathepsin B induces apoptosis in CD8+ T lymphocytes, preventing them from becoming memory cells (64).
**B lymphocytes**	
Antigen presentation and homeostasis	Cathepsin S is important for Ii cleavage and antigen presentation in B lymphocytes (26). Cathepsin L negatively regulates B lymphocyte production in bone marrow and restricts numbers of peripheral B lymphocytes (65). Cathepsin B mediates pro-B lymphocyte programmed cell death induced by CpG TLR-9 (66).

#### In the Cytosol

Cytosolic cathepsins are primarily involved in the control of cell death or survival. Various external and internal stimuli, such as reactive oxygen species (ROS), lysosomotropic agents, certain chemotherapeutics, etc. lead to destabilization of lysosomes and provoke lysosomal membrane permeabilization. Leaked cathepsins (B, H, L, K, and S) trigger apoptosis by activating pro-apoptotic Bid and by degrading anti-apoptotic Bcl-2 family proteins, consequently promoting release of cytochrome C from mitochondria and engaging caspases (67). Cathepsins B and L mediate supraoptimal, activation-induced apoptosis in lymphocytes, ensuring that potential autoreactive cells are rapidly removed (68, 69). Autophagy, the other process that regulates cell fate, is a universal response to cell stress due to nutrient, energy, or growth factor deprivation or to hypoxia. The final outcome of the autophagic process, cell death or preservation is, in great part, regulated by lysosomal peptidases. In general, cysteine cathepsins S, B, L, and C exhibit pro-survival properties. Inhibition of their activity disturbs autophagic protein turnover and results in the accumulation of autophagosomes and generation of oxidative stress. Excessive oxidative stress eventually overcomes cell adaptive mechanisms and tips the balance toward apoptosis (70, 71). To add another layer of complexity, cathepsins are even more widely involved in the intracellular signaling pathways. A striking example is the exopeptidase cathepsin X that, by cleaving neurotrophic factor y-enolase, disconnects MAPK/ERK and PI3K/Akt survival signaling in neuronal-like cells (72, 73).

#### In the Nucleus

Alternatively, some cathepsins may enter the nucleus. For example, the cathepsin L shorter isoform, devoid of an NH_2_-terminal signal sequence, has been shown to process the transcription factor CDP/Cux that controls transcription of genes associated with cell proliferation and differentiation (74). Another way in which cathepsin L can regulate gene expression is by cleaving the tail of histone H3 (75). Further, interaction in nucleus between cathepsin S and tumor suppressor protein BRCA1 has been observed in breast cancer cells. Cathepsin S cleavage facilitates ubiquitin-mediated degradation of BRCA1, abolishing its DNA-repair activity, and thus contributing to genomic instability in cancer cells (76). Normally, cathepsin L nuclear activity can be counterbalanced by its endogenous inhibitor stefin B, as shown in normal intestinal epithelial cells. However, in colorectal carcinoma cells, stefin B is displaced from the nucleus, coinciding with enhanced proteolytic activity of cathepsin L and increased cell proliferation (77). Indeed, apart from differential compartmentalization and their synthesis as zymogens, endogenous inhibitors constitute yet another important level of control over the activity of cathepsins.

## Endogenous Cathepsin Inhibitors and Their Significance in Regulating the Immune System

### Type I Cystatins

As in other cells under physiological conditions, the activity of cysteine cathepsins in immune cells is controlled by endogenous cysteine protease inhibitors. Cystatins constitute an evolutionarily related family of reversible, tight-binding inhibitors of lysosomal cathepsins belonging to the C1 family. Some cystatins also inhibit asparaginyl endopeptidase (AEP; legumain) from the C13 family through other binding site. In addition, they protect their host from the harmful effects of cysteine peptidases from microorganisms and parasites. Cystatins are classified according to their structural similarities, the number of cystatin-like domains and the presence of disulfide bridges (78). Type I cystatins, *stefins A and B*, are mainly intracellular non-glycosylated polypeptides without disulfide bridges (78, 79). There are not many reports of the involvement of these two stefins in immune system processes. Stefin A is abundant in follicular dendritic cells in germinal centers, presumably involved in preventing B cell apoptosis (80). Stefin B is upregulated in human monocytes after exposure to lipopolysaccharide (LPS), suggesting its anti-inflammatory function (81). The latter was confirmed, in another study that showed increased production of nitric oxide, in parallel with decreased synthesis of IL-10 in bone marrow-derived macrophages (BMDM) from stefin B deficient mice (82).

### Type II Cystatins

In contrast to stefins, type II cystatins contain two conserved disulfide bridges and a signaling sequence that enables them to be secreted to the extracellular space. They are mostly non-glycosylated, with the exception of cystatins E/M and F. *Cystatin F* is an exception in that it is the only cystatin that targets cathepsins inside endosomes and lysosomes (83). Further, cystatin F is expressed predominantly in immune cells, hence known as leukocystatin. After its synthesis, most of the cystatin F is retained intracellularly, being sorted to the endolysosomes via the mannose-6-phosphate receptor pathway (78, 79). It is synthesized as an inactive disulfide-linked dimer that has to lose 15 amino acid residues at the N-terminus (presumably cleaved by cathepsin V) to be converted to the active monomer. Truncated monomeric cystatin F is a potent inhibitor of cathepsins C and H (84), the latter known as major progranzyme convertases that direct the cytotoxicity of NK cells and cytotoxic T lymphocytes (CTL) (85). The implications of cystatin F as a regulator of immune cell cytotoxicity will be discussed in detail later. In myeloid cells, the levels and localization of cystatin F correlate with the stage of differentiation. In immature DC, cystatin F is co-localized with cathepsin S in the Golgi apparatus whereas, in mature, adherent DC it is translocated toward the lysosomes and interacts with cathepsin L (86). Transition to the adherent state is one of the crucial events during DC maturation. It is facilitated by another cysteine peptidase, cathepsin X (40). Cathepsin X is not inhibited by cystatin F, however, since cathepsin L is needed to activate procathepsin X, it is tempting to speculate that cystatin F, as a cathepsin L inhibitor, indirectly controls cathepsin X dependent adhesion, and the maturation of dendritic cells (86). Later, it was resolved that cystatin F expression is controlled dynamically by transcription factor C/EPB α (87). Whereas monocyte-derived dendritic cells express cystatin F (86), the differentiation of monocytes to granulocytes and macrophages (88) is marked by decreased cystatin F expression, since C/EPB α does not bind cystatin F promoter (87).

The other, and most intensively studied, type II cystatin, *cystatin C*, is ubiquitous and acts as a potent, nano- and pico-molar inhibitor of cathepsins B, H, L, and S (89). Combined activation of hematopoietic cell-specific transcription factors IRF8 and PU.1 promotes high expression of cystatin C in mouse macrophages and in a subpopulation of CD8+ dendritic cells (90). Expression, localization and secretion of cystatin C vary according to the maturation stage of human dendritic cells. However, cathepsins S, L, and H, which are all inhibited by cystatin C, are found in separate cell compartments and can only be bound by their inhibitor when in the extracellular space. In some instances, protease inhibitors stabilize rather than abolish peptidase activity. Cystatin C could therefore stimulate proteolysis in order to facilitate migration of DC to the lymph nodes (91). By comparing serum cystatin C levels between irradiated cystatin C +/+ mice that were transplanted with bone marrow from either cystatin C null or wild type mice, it was shown that immune cells actually contribute a large proportion of the serum cystatin C (~30%) (92). Immune cells are thus an important source of cystatin C, especially when recruited to the site of inflammation. What is more, under the influence of ROS, intracellular cystatin C readily dimerizes via a process known as domain swapping (93). Cystatin C dimers lack an inhibitory function but can aggregate further to form toxic amyloid deposits. During pathological events that lead to increased oxidative stress, dimeric cystatin C can be released from macrophages or dendritic cells undergoing apoptosis, thus exacerbating tissue damage (92). Extracellular cystatin C also binds TGF-β, an important immunomodulatory cytokine, and prevents TGF-β signaling by blocking access to its receptor (94). These findings indicate that cystatins possess active sites and regulate functions that are unrelated to proteolysis.

### Type III Cystatins

Type III cystatins, termed *kininogens*, are high molecular weight glycosylated proteins that contain three tandemly repeated cystatin type-II like domains and eight disulfide bridges. Kininogens participate in the activation of innate immunity through release of the inflammatory mediator bradykinin (78). Moreover, some *serpins*, typically serine peptidase inhibitors, can inhibit the functions of caspases and cathepsins through cross-class inhibition (95). Serpin Spi2A (also termed SpiA3G) was identified by a genetic screen as a prime candidate for assigning commitment of cytotoxic CD8+ T lymphocytes to a memory lineage. In experiments with retrovirally transduced bone marrow chimeras, overexpression of Spi2A resulted in a lower incidence of programed cell death among antigen specific CTL. In reverse, Spi2A antisense mRNA triggered CTL apoptosis in chimera mice. It is postulated that cytoplasmic Spi2A, through inhibition of cathepsin B, prevents programmed cell death in CTL committed to become memory cells (64, 96). In addition, Spi2A could regulate cathepsin L activity as they both were found to co-localize in nuclei of RAW 264.7 mouse macrophages. However, it should be noted that Spi2A is a mouse specific serpin with no human homolog found to date (97).

### Thyropins

Another group of cysteine cathepsin inhibitors, termed thyropins, was identified with the discovery of *p41 invariant chain (Ii) fragment*. Even though thyropins are functionally unrelated proteins, they have, in common, homology to thyroglobulin type-1 domains (1). p41 is one of the four isoforms of the chaperone molecule Ii, which associates with major histocompatibility complex (MHC) II molecules in antigen-presenting cells. p41 Ii guides the proper folding of MHC II α/β heterodimers in the endoplasmic reticulum and prevents premature peptide loading by occupying the peptide-binding groove. The invariant chain also contains the signaling motif that directs MHC II molecules to the endosomal pathway. Cysteine peptidases sequentially cleave Ii and another chaperone molecule HLA-DM assists displacement of the remaining CLIP fragment by antigenic peptides (27). Dendritic cell endogenous p41 has been shown to stabilize the mature form of cathepsin L in late-endocytic compartments (98). In contrast, exogenously supplied p41 inhibited cathepsin L activity in immature mouse bone-marrow-derived DC and impeded secretion of IL-12 after activation (99). DC were shown to secrete active cathepsin L in complex with p41 fragment in response to inflammatory stimuli. This contributes to local increase in ECM proteolytic activity and could be relevant for enhanced migration and recruitment of APC (100). The repertoire of proteases inhibited by p41 fragment has been extended further to cathepsins F, H, K, S, and V (101). To summarize, p41 fragment is a strong modulator of antigen presentation through the control of cathepsin activity.

Although thyropins were named after the structure of the thyroid hormone precursor, thyroglobulin, its 11 thyroglobulin type-1 domains act as substrates, rather than inhibitors of cysteine cathepsins. Nevertheless, besides p41, some other inhibitory thyropin members can be found in mammals. Brain cells secrete proteoglycans *testicans* (testican and its homologs −2 ad −3) with yet unknown functions, possessing inhibitory activity toward cathepsin L. *Nidogens* (1 and 2), produced by mesenchymal cells, are necessary constituents of basement membranes since they link laminins and type IV collagens non-covalently (102), and have been shown to inhibit cathepsin K (103). However, at higher concentrations of the enzyme, testicans switch from being cathepsin L inhibitors to cathepsin L substrates (104) and nidogen-1 is prone to proteolytic degradation by cathepsin S (105).

### The Role of Cathepsins in Tumor Diagnosis and as Targets for Therapeutic Intervention

Numerous studies established a prominent link between cysteine cathepsins and tumor progression. The protein levels and in particular increased activity of these peptidases were correlated with poor prognosis and high tumor grade in different tumor types (106, 107). Accordingly, cathepsins received considerable attention as therapeutic targets, resulting in development of several small molecular inhibitors. JPM-OEt, a cell permeable derivative of epoxysuccinyl compound E64, was one of the first broad spectrum inhibitors which successfully withstanded trial in pre-clinical model of Rip1-Tag2 model of pancreatic islet cancer. However, due to its poor bioavailability the results could not be reproduced in polyoma middle T oncogene-transgenic breast cancer mouse model (108). Testing several other irreversible broad spectrum inhibitors rose concerns regarding possible side effects of long-term systemic ablation of cysteine cathepsins encouraging design of specific and reversible inhibitors (109). To date the only selective inhibitor to reach phase III clinical trials has been monoclonal antibody odanacatib, indicated for blocking the harmful activity of cathepsin K in breast and prostate cancer bone metastasis. It was later discontinued owing to unwanted on-target effects resulting in increased cardiovascular adverse events (110). When taking into account redundancy and compensatory mechanisms among cysteine cathepsins, the greatest therapeutic potential of their inhibition is expected to be its use in combinational therapies (e.g., in conjunction with immuno- and radiotherapy).

Nonetheless, abundant cathepsin activity in extracellular space of TME and in infiltrating immune cells can also be exploited for targeted drug delivery or for detection of cathepsin-directed imaging probes. In recent years several innovative approaches have been developed for both applications and some of them have already been adopted in clinical practice. Such example is FDA approved therapeutic Adcetris® (brentuximab vedotin), an antibody-drug conjugate containing cathepsin-sensitive peptide linker, enabling selective release of cytotoxic payload into the tumor tissue (111, 112). However, efficacy of antibody-drug conjugates could be hindered by low antigen expression on tumors and off-target effects on healthy tissues. In order to improve targeting Probody™ technology was introduced, whereby peptidases serve to remove peptide mask that blocks antigen recognition site on monoclonal antibody (107, 113). PD-L1- targeting Probody CX-072 has already advanced from proof of concept to the ongoing phase II clinical trial. Additionally, cathepsin cleavable linkers could be used for delivery of drug-containing nanoparticles and small molecule pro-drugs (112). The need for better understanding of cysteine cathepsins function, dynamics and localization in tumors led to development of small molecule reporters of their activity. Activity based probes proved to be powerful diagnostic tools for non-invasive imaging and residual-tumor detection during surgery. LUM-015, comprising of fluorescence quencher molecule attached through tetrapeptide GGRK to polyethylene glycol linker and Cy5 fluorophore, was the first pan-cathepsin imaging probe to be translated from bench to bedside (114).

## Cysteine Cathepsins in Myeloid Cells—Maintaining Immune Cell Homeostasis

### Antigen Presentation

Recent discoveries shifted the perspective of cysteine cathepsins as promoters of tumor invasion and migration to their immunomodulatory function in cancer (115). The most studied to date is their role in controlling MHC II-dependent antigen presentation. For priming of naïve T lymphocytes, a prerequisite step in tumor eradication, tumor antigens need to be internalized by DC, processed and displayed on MHC class I and II molecules to T cell receptors. These two different pathways of antigen presentation are employed to engage cytotoxic CD8+ or helper CD4+ lymphocytes, respectively. Antigens that are present in the cytosol are degraded by proteasome and transferred to endoplasmatic reticulum by transporter associated with antigen processing (TAP) to join with MHC I molecules. Alternatively, antigens can be degraded in late phagolysosomes to become MHC II epitopes. Cysteine cathepsins have been shown to be important in cross-presentation and, especially, in MHC II-dependent presentation [reviewed in (25) and (26)]. They generate immunogenic peptides through limited proteolysis and importantly, regulate trafficking of both MHC I (116) and II molecules by cleaving the invariant chain (117). Although *in vitro* several cysteine peptidases have been shown to be capable of processing the Ii, genetic ablation of specific cathepsins in mice revealed an essential role in cleaving Ii for cathepsin L in cortical thymic epithelial cells and for cathepsin S in DC, macrophages and B cells (27).

### Antigen Processing

In addition to antigen loading on MHC II, cysteine cathepsins regulate antigen processing. The impact of cysteine peptidase activity on MHC II epitope generation has therefore been extensively studied. Loss of activity of a particular enzyme could significantly influence the CD4+ epitope repertoire in *in vitro* models. However, experiments *in vivo* failed to demonstrate that presentation of a particular antigen depends on a specific cathepsin. It appears that epitope functionalities in conventional antigen presenting cells are determined rather by the overall peptidase activity and by their relative distribution through the endosomal compartments (24, 25). The exception is the MHC II/peptide repertoire presented by cortical thymic epithelial cells; this is reduced in the absence of cathepsin L (118). Nonetheless, cathepsin S stands out as a crucial cysteine peptidase in generating peptides for TAP-independent cross-priming of T lymphocytes through MHC I (119). The importance of cathepsin S for antigen presentation is further supported by the findings that the anti-inflammatory cytokine IL-10 suppresses IFN-γ-induced MHC II and cathepsin S expression in macrophages (120). More importantly, IL-10 prevents upregulation of cathepsins S and B activities in DC (121). Of note, IL-10 is known to be involved in generating tolerogenic DC that, instead of activation, induce T lymphocyte anergy (122, 123). In contrast, proinflammatory cytokines, such as IFN-γ and TNF-α, increase cathepsins S and B activities and the surface display of MHC II/peptide complexes in DC (121, 124). Elevated cathepsin S activity in activated DC could be, at least in part, attributed to reduced endolysosomal levels of its endogenous inhibitor cystatin C (125).

In order to achieve proper antigen processing while, at the same time, prevent its destruction, lysosomal proteolysis in APC needs to be stringently controlled. Indeed, cathepsins are much more abundant in macrophages than in DC and B cells, corresponding to the poorer recovery of engulfed antigen in the former cell type (126). DC employ several mechanisms to control peptidase activities during maturation. After phagocytosis of foreign material, NADPH oxidase 2 (NOX2) changes vesicular redox potential, thereby inactivating cysteine cathepsins in DC (127, 128). Contrary to the NOX2 function, enzyme gamma-interferon-inducible lysosomal thiol reductase (GILT) can maintain cathepsins in their reduced-active-state. Phipps-Yonas et al. showed that GILT diminishes cathepsin S protein levels in primary B cells and can also disrupt the tertiary structure of cathepsin S by breaking its disulfide bonds (129). In contradiction to these observations, Balce et al. reported that GILT was necessary for maintaining the proteolytic efficacy of cathepsin S in phagosomes of macrophages, especially after NOX2 activation (130). In addition, the extent of proteolysis is regulated by maturation-dependent pH change in endosomes of APC. In immature DC, low proteolytic rates support the conservation of immunogenic peptides, allowing them to remain intact for several days until DC reach lymph nodes (131). Only after receiving maturation stimuli does the ATP-dependent vacuolar proton pump, V-ATPase, lower the lysosomal pH, allowing for effective MHC II antigen processing (131).

A recent study on tumor cells revealed that lysosomal V-ATPase activity is enhanced by the cytosolic transcription factor signal transducer and activator of transcription-3 (STAT-3). STAT-3 is a pleiotropic transcription factor, recognized as an oncogene in various cancers. Phosphorylation of tyrosine residue 705 governs its function as a transcription factor, although other post-translationally modified forms have been shown to control cell metabolism and migration as well, acting independently on transcriptional regulation. Tumor-derived factors propagate persistent activation of STAT-3 in immune cells; this is manifested in immature phenotype and immunosuppressive characteristics of DC and monocytes (132). STAT-3 is able to induce expression of cysteine cathepsins, possibly in cooperation with another cancer-associated transcription factor, hypoxia-inducible factor-1-alpha, HIF-1α. Recently, hypoxia response elements have been recognized in the promoter region of the cathepsin B gene; cathepsin B was confirmed as the HIF-1α target gene (133). In addition, STAT-3 synergizes with STAT-6 signaling to activate inositol-requiring enzyme 1α, IRE1α, which is an important mediator of unfolded protein response to endoplasmic reticulum stress. Genetic deletion of both STATs or pharmacological inhibition of IRE1α provoked pronounced reduction in secretion of cathepsins B, L, S, and X from IL-4 and IL-6 stimulated murine BMDM (134). In contrast to the mouse data, IL-4/IL-6 dual stimulation of human monocyte-derived macrophages elicited a completely different set of genes, none of them coding for cysteine cathepsins (135). Such discrepancies between mouse and human models must be taken into consideration; they make comparisons between studies using different sources of immune cells problematic (136). Alterations in the profile of cysteine cathepsins in cancer re-shape immunity in such a way as to foster tumor progression and STAT-3 appears to mediate the crosstalk between cancer cells and the immune system.

### Toll-Like Receptor Activation and Cytokine Secretion

The role of cysteine cathepsins in maintaining immune cell homeostasis goes well-beyond the canonical regulation of antigen presentation. Cathepsins are indispensable for activation of innate immune cells by engaging their intracellular endolysosomal toll-like receptors (TLR). Ectodomains of transmembrane TLR-3,−7, and−9 are cleaved and trimmed in a two-step process that involves first AEP and then cysteine cathepsins (B, F, H, L, or S) (31–33). As in antigen presentation, redundancy among cysteine cathepsins ensures that TLR can be activated across various cell types (31). Conversely TLR signaling augments cathepsins' activities through a positive feedback loop. Treating mouse macrophages with different TLR ligands (LPS, poly I:C or peptidoglycan) induced differential increase in proteolytic activities of cathepsins B, L, and S. Moreover, increase in cathepsin activity could be transferred to bystander cells through cytokines (IL-1β, TNF-α, IFN-β) in conditioned medium from TLR-stimulated cells (137). Inversely, cathepsins can modify cytokine secretion independently of their role in TLR activation. Bone marrow-derived DC and macrophages from cathepsin B null mice were found to produce more IL-12 in response to *Leishmania major* infection (35). However, in a similar study IL-12 mRNA expression did not differ between wild type and cathepsin B null mice (138). Another study showed that cathepsin K contributed to maximal induction of pro-inflammatory cytokine IL-6 and that cathepsin L enhanced non-oxidative killing activity in murine macrophages infected with *Staphylococcus aureus* (34). More prominent is the relationship between cathepsin B and excessive production of IL-1β. Cytokine IL-1β is considered to be a key proinflammatory driver in Alzheimer's and Parkinson's diseases, multiple sclerosis, atherosclerosis, type I diabetes (36) and cancer (37). Cytosolic cathepsin B activates NLRP3 inflammasome through an as-yet unidentified mechanism followed by caspase-1 conversion of pro-cytokines IL-1β and IL-18 into biologically active forms (36, 37). Several studies have confirmed the beneficial effect of cathepsin B inhibition by reducing IL-1β-fueled inflammation (139–141), although contributions by other members of the cathepsin family should not be underestimated (142).

### Autophagy—Myeloid Cell Survival and Differentiation

Autophagy plays diverse roles in maintaining immune cell homeostasis and is closely related to the lysosomal function (143). In a study by Qi et al. cathepsin B genetic ablation in murine BMDM resulted in a nearly 2-fold increase in the number of lysosomes as well as in enlargement of both single-membrane lysosomes and double-membrane autophagosomes compared to wild type cells, clearly showing increased autophagy. Mechanistically, cathepsin B cleaves the lysosomal calcium flux channel TRPML1, thus preventing release of calcineurin from the lysosomes and engagement of transcription factor TFEB. In murine macrophages infected with *Francisella novicida*, genetic deletion or pharmacological inhibition of cathepsin B therefore enhanced lysosomal biogenesis, fusion with phagosomes and enabled effective bacterial clearance (28). Of note, TFEB is also involved in controlling MHC II presentation by contributing to lysosome acidification after phagocytosis and by increasing transcription of cathepsin genes, while at the same time inhibiting the cross-presentation process on MHC I molecules (144). Furthermore, induction of autophagy is an important mechanism for regulating immune cell survival and differentiation. Blood circulating monocytes are short lived cells that die of apoptosis in 24–72 h in the absence of activating stimuli. Blocking lysosome-phagosome fusion with chloroquine during macrophage differentiation promotes programmed cell death (29). Accordingly, monocyte differentiation to macrophages is marked by an overall increase in cathepsin activity. GB11-NH_2_, a small molecule inhibitor of cysteine cathepsins B, L and S, reduces the viability of murine BMDM during both classical, M1, and alternative, M2, differentiation, due to disruption of autophagy and to increased oxidative stress levels. These results were substantiated in 4T1 tumor bearing mice, where cathepsin inhibition provoked apoptosis of tumor-associated macrophages (TAM). Strikingly, even though the inhibitor did not affect tumor cell viability *per se*, the death of stromal TAM indirectly caused apoptosis of neighboring tumor cells (70). These results are in accordance with other reports, demonstrating that TAM depletion reduced tumor burden (145–147). After differentiation, regulation of autophagic flux by cathepsin S was found to be necessary for maintaining TAM in an M2 polarized state in the tumor microenvironment (30).

### Integrin Regulation

Immune cells are subjected to distinct morphological and cytoskeleton structure changes during differentiation (148, 149). Endopeptidases like cathepsin B, L, or S facilitate leukocyte migration through proteolysis of ECM. However, the exopeptidase cathepsin X has a unique role in immune cell adhesion and migration due to its ability to interact with integrin receptors. The integrin family consists of 24 types of heterodimers that are formed from the combination of 18 α-subunits and 8 β-units. Integrins recognize different sets of extracellular ligands and serve as transmembrane adhesion receptors with bidirectional signal transduction capability. In order to bind ligands, integrins need to switch from a bent, low affinity state to an extended conformation (150). Signals from chemokine, T or B cell receptors can trigger “inside-out” signaling that promotes the binding of adaptor molecules (talins, kindlins, vinculins, actinins) to the cytoplasmic domains, inducing the conformational changes required for integrin activation. On the other hand, extracellular ligand binding, with clustering, or fluid shear force, can propagate alterations to the cytoplasmic regions from “outside in” signaling. Both modes of integrin activation and affinity regulation are closely interlinked (151, 152). The cytoplasmic tails of α and β subunits are essential for integrating complex signaling networks as they coordinate the binding of structural, scaffolding, and signaling proteins, thereby influencing cell polarity, motility, proliferation and differentiation (153). Phosphorylation, as well as proteolytic modification of integrin tails, modulates integrin association with their intracellular binding partners (153).

Cathepsin X contains RGD and ECD integrin recognition elements in its pro-enzyme and mature forms, respectively (154, 155). Heparan sulfate, which is involved in integrin regulation, can anchor extracellular cathepsin X to the cell surface, influence cathepsin X catalytic activity and even promote cell uptake of cathepsin X (156). The observation that the active form of cathepsin X interacts with integrin β_2_ subunit is of particular importance, since this group of integrin receptors can be found only in leukocytes (157). Mice genetically deficient in integrin β_2_ subunit display features of leukocyte adhesion deficiency that result in severe immune deficits, including leukocytosis, spontaneous infections, impaired neutrophil recruitment and reduced T lymphocyte proliferation (158). As shown by our group, cathepsin X sequentially cleaves four amino acid residues from the C-terminus of β_2_ cytoplasmic tail, allowing integrin transition from extended, closed conformation with intermediate affinity to the open conformation with high affinity for ligand binding (53, 54). Several *in vitro* models were used to demonstrate the importance of cathepsin X in the regulation of integrin function. Inhibition of cathepsin X activity abolished the adhesion to fibrinogen of differentiated U937 cells via Mac-1 (integrin α_M_β_2_) receptor, while addition of recombinant cathepsin X restored it. Phagocytosis, another Mac-1 dependent process in macrophages, was also impaired (39). Moreover, adhesion through a Mac-1 receptor is essential for effective DC maturation. After maturation stimuli, cathepsin X has to be translocated to the plasma membrane to enable Mac-1 activation and podosome formation. In the absence of cathepsin X activity, DC are dysfunctional, with reduced ability to secrete cytokines and to stimulate lymphocyte proliferation (40).

Cathepsin X fine-tunes the activity of another β_2_-chain integrin, lymphocyte function-associated antigen-1 (LFA-1). LFA-1 reveals an opposing role to Mac-1 in regulation of lymphocyte activation. Activation of LFA-1 enhances the formation of uropods, i.e., cell extensions that enable migration of T lymphocytes (50). Also, the interaction of LFA-1 with intercellular adhesion molecules, ICAMs, is required for the formation of stable immunological synapses between T lymphocytes and APC. On the other hand, Mac-1 engagement on mature APC inhibits antigen presentation and limits T lymphocyte activation (52, 159). Indeed, the constitutive activity of Mac-1 receptor on macrophages explains why the latter are such poor stimulators of T lymphocytes (159). Cathepsin X-mediated regulation of integrin receptor activity has been shown to be of clinical relevance in patients infected with *Helicobacter pylori*. Therapy resistant bacterial strains induced membrane co-localization of cathepsin X and Mac-1 in THP-1 cells (160). Inhibition of cathepsin X prevented internalization of TLR-2 and TLR-4 and lowered the production of cytokines IL-1β, IL-8, IL-10, IL-6, suggesting that cathepsin X localization and activity impacts the efficacy of the immune response (161). In a recent study it was demonstrated that deletion of cathepsin X in non-small cell lung cancer cells prevented β_3_ integrin activation and abrogated signaling downstream the focal adhesion kinase (162). All these data designate cathepsin X as a crucial regulator of integrin signaling.

In addition to cathepsin X, another cysteine cathepsin, cathepsin H, regulates integrin-cytoskeleton linkage. Cathepsin H, as a monoaminopeptidase, processes talin at its N-terminal head domain that contains the integrin binding site. It has been shown to co-localize with talin in focal adhesions at the leading edge of migratory cancer cells. Silencing of cathepsin H in prostate cancer cells reduced migration and promoted activation of α_ν_β_3_ integrin, thus confirming the biological relevance of cathepsin H interaction with talin (163).

### Chemotaxis

Cysteine cathepsins also influence immune cell trafficking by processing chemotactic cytokines with the CXC motif. Cathepsins B, K, L, and S generate active forms of angiogenic ELR-CXC chemokines that recruit neutrophils, and inactivate angiostatic non-ELR CXC chemokines capable of attracting T lymphocytes (20). Interestingly, tumor breast cancer cells have been shown to upregulate and secrete more cathepsin B on stimulation of chemokine receptor CXCR3 with non-ELR chemokines CXCL9 and CXCL10. In that way cancer cells employ cysteine cathepsins to reduce lymphocyte infiltration (164). While other CXC members are inflammation inducible cytokines, chemokine CXCL12 is constitutively expressed and is important for retaining hematopoietic stem cells in their niches. Osteoclasts secrete several cathepsins that digest CXCL12, but only cathepsin X is able to reduce attachment of hematopoietic stem cells to the osteoclasts (41). Of note, CXCL12 (also designated stromal cell-derived factor-1, SDF-1) is frequently overproduced in tumor microenvironments where it assists the accumulation of myeloid-derived suppressor cells (see below) (165).

## Cysteine Cathepsins in Tumor-Associated Myeloid Cells—Regulating Immunosuppression

**Figure 1:**
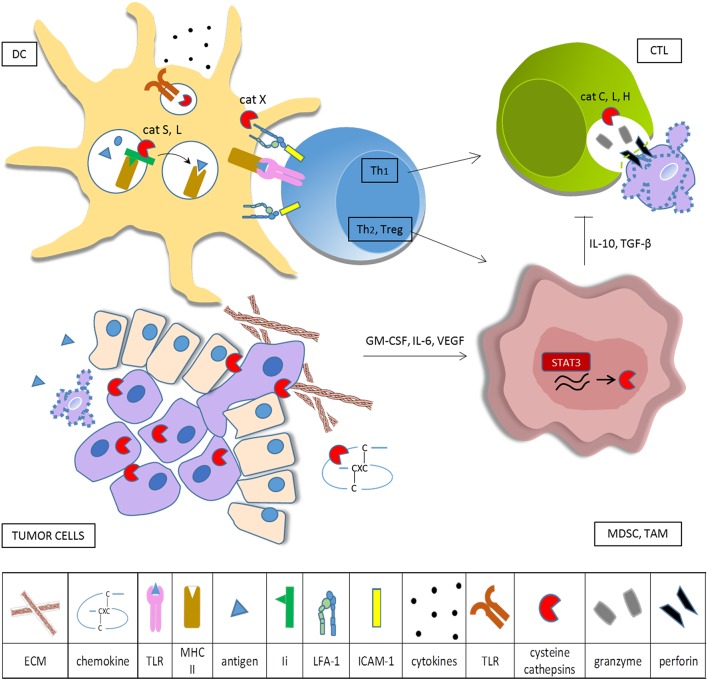
Cysteine cathepsins in tumor-associated immune cells. Tumor cells overexpress cysteine cathepsins in order to enhance their survival, proliferation, motility, and invasive potential. When secreted into the extracellular space, cysteine cathepsins remodel ECM and degrade CXC chemokines. Tumor cells produce cytokines that skew myeloid cells toward the immunosuppressive phenotype and constitutively activate transcription factor STAT3, resulting in increased synthesis of cysteine cathepsins in TAM and MDSC. In DC cathepsins, L and S are required for effective antigen presentation on MHC II and cathepsin S is involved in cross presentation on MHC I. Cathepsins also influence DC cytokine production and secretion, partly through regulation of TLR activation. Cathepsin X modulates the affinity of integrin β2 receptors, thus controlling cytoskeleton rearrangements and LFA-1 dependent signaling during immunological synapse formation. Primed helper T cells can either stimulate (Th1) or deteriorate (Th2, Treg) anti-tumor immunity. CTL make use of the perforin-granzyme pathway for cancer cell killing, cathepsins L, C, and H are needed for activation of pro-perforin and pro-granzymes. Immunosuppressive myeloid cells impede cytotoxic cell activity.

Recent studies in colorectal carcinoma, melanoma, clear cell renal cell carcinoma and ovarian cancer have highlighted the importance of assessing density, composition, functional state and localization of tumor immune infiltrate to predict clinical outcome and response to treatment. Based on their immune profiles tumors can be stratified as immunogenic, immune neglected or inflammatory, corresponding to good, intermediate or poor prognosis, respectively. The first tumor type is characterized by high antigenicity that promotes antigen presentation, anti-tumoral M1 macrophage polarization, T cell activation and recruitment. In contrast, tumors in the second category downregulate their MHC I molecules, produce little cytokines and are devoid of lymphoid and myeloid infiltrates. Finally, tumors of the third type are marked by expression of genes involved in myeloid cell chemotaxis, angiogenesis, inflammation, and immunosuppression that prevents proper T cell education (166, 167). Myeloid cells, a major component of TME, are actively involved in bidirectional interactions with tumor cells. In order to coordinate immune response, they need to be extremely plastic, which also means they are easily subverted to regulatory phenotype under the influence of tumor-derived factors. Chronic inflammatory conditions promote myeloid cell recruitment to the tumor site where tumor-derived cytokines such as IL-10, TGF-β, GM-CSF, IL-6 initiate their immunosuppressive properties (168–170). Recent studies indicate that myeloid regulatory cells have profound effect on the course of the anti-tumor immune response and effector functions of adaptive immune cells (summarized in Figure 1). Despite efforts to categorize different subsets of tumor-associated myeloid cells, lack of specific cell markers still represents a challenge to a better understanding of their molecular and functional signature.

### Tumor-Associated Macrophages and Myeloid-Derived Suppressor Cells

TAM constitute the major leukocyte population in the tumor tissue and their number has been correlated to poor prognosis in many solid human cancers (171). Pro-tumoral macrophages are usually described as M2 or alternatively polarized subtype (172, 173) and, in humans, designated by cell marker CD68 in addition to CD163 and CD204 (174). However, this view is over simplistic as it covers only the highly extreme state in the macrophage polarization spectrum (175, 176). In addition to converting macrophages toward tumor-promoting phenotype, cancer drives the expansion of a heterogenic immature population, known as myeloid-derived suppressor cells (MDSC). MDSC has been recognized as a vital player in immunosuppression due to their ability to potently reduce T cell effector functions (177, 178). MDSC are divided, according to their similarity to normal monocytes and granulocytes, into monocytic, Mo-MDSC, and polymorphonuclear or granulocytic, PMN-MDSC or G-MDSC, subsets (179). Mo-MDSC are defined as HLA-DR^−/lo^, CD33^+^, CD14^+^ cells in humans and CD11b^+^Gr1^hi^ LY6C^+^ cells in mice. Correspondingly, PMN-MDSC are CD14^−^ CD11b^+^ CD33^+^ CD15^+^/CD66b^+^ or CD11b^+^ Gr1^hi^ Ly6G^+^ cells in humans or mice, respectively (179, 180). MDSC represent an independent prognostic marker in solid tumors and their frequency correlates negatively with the clinical outcome (181). MDSC and TAM are closely connected and engaged in cross-talk that further skews macrophages toward M2 phenotype (182). MDSC can also be detected in patients with chronic infections, autoimmune diseases and obesity (183). However, their function is not always harmful; for example they are needed for establishing feto-maternal tolerance in pregnancy (184, 185) and MDSC-based cell therapy has been considered a promising strategy for improving allograft survival in transplanted recipients (186).

### TAM and MDSC Immunosuppressive Properties

The development of TAM and MDSC is governed by a series of transcription factors, of which signaling through STAT-3 and NF-κB is essential (187). In addition, MDSC readily differentiate to TAM in the tumor site (188, 189). It is therefore not surprising that TAM and MDSC share many of their immunosuppressive functions (169, 175). They express the enzymes arginase 1 (Arg1), inducible nitric oxide synthase (iNOS) (190) and indoleamine-2,3-dioxygenase (IDO) (191) that interfere with arginine and tryptophan metabolism, which is detrimental for T lymphocyte proliferation. iNOS also generates nitric oxide that disrupts the IL-2 receptor signaling pathway (192). In addition, reactive oxygen and nitrogen species are generated by combined action of iNOS, Arg1 and NOX2. Peroxynitrite, through nitrosylation of certain lymphocyte chemoattractants, prevents tumor homing of T lymphocytes. Moreover, nitrosylation/nitration of T lymphocyte receptors impairs antigen recognition (193). Induction of cyclooxygenase 2 (COX2) enables generation of the immunosuppressive mediator prostaglandin E2 (PGE2) (194). Tumor-educated myeloid cells also secrete cytokines TGF-β and IL-10 which, on the one hand, induce expansion of regulatory T lymphocytes (Treg) and, on the other, impact negatively on NK cell and DC activities (195). Furthermore, they express inhibitory ligands, such as PD-L1, that mediate T-cell dysfunction (196). MDSC or TAM also support cancer progression through non-immune mechanisms. They enhance cancer cell stemness (197, 198) and resistance to chemotherapy (199), release proangiogenic peptides necessary for angiogenic switch (200), facilitate tumor intravasation into the circulation and assist metastatic process by creating a favorable environment at premetastatic sites as well as by inducing the tumor cell epithelial to mesenchymal transition (201, 202).

### Myeloid Cells as Major Contributors to Cysteine Cathepsin Activity in Cancer

Myeloid cells (TAM, MDSC) are important contributors to increased levels of cysteine cathepsins either in tumor tissue or in bodily fluids. It has been shown that general cathepsin activity in RT2 pancreatic islet cancer and PyMT breast cancer mouse tumors emanates mostly from TAM (203). This observation led Park et al. to propose a scoring system for assessing invasion risk in breast cancer patients. Patient monocyte-derived macrophages secrete different amounts of active cathepsins and their endogenous inhibitor cystatin C. In line with the inherent patient-to patient variability, macrophage-assisted *in vitro* migration of MDA-MB-231 breast cancer cell line correlated positively with higher ratio of active cathepsins vs. cystatin C levels (204). Hence proteolytic profiling in myeloid cells could help identify individuals with increased invasive cancer phenotype. The contribution of immune cell-supplied cathepsins on tumor progression has been evaluated in several studies. It is now well-established that monocytic cells upregulate cathepsin expression on interaction with tumor cells (205, 206). Further, cysteine cathepsins expressed by tumor and stromal cells display disparate functionalities. In mouse pancreatic neuroendocrine tumor model (PanNET), cancer-cell intrinsic cathepsin X supports tumor proliferation, while macrophage-secreted cathepsin X facilitates cancer cell invasion through RGD-dependent binding of integrin receptors (207). In a mouse model of hereditary polyposis, NIR live imaging revealed abundant cathepsins' activity in macrophages and MDSC, infiltrating the lesions. The fluorescent probe (ProSense 680) that was used is preferentially activated by cathepsin B but can be hydrolyzed by other cathepsins and related peptidases. In this particular model, MDSC depended critically on cathepsin B activity, since mice deficient in cathepsin B failed to accumulate MDSC. Interestingly, anti-TNF-α treatment comparably reduced MDSC density and caused a significant drop in the activity of polyp specific cathepsin B (46). An earlier study already demonstrated that cathepsin B deficiency abrogated trafficking of TNF-α containing vesicles to the cell membrane by using three different *in vitro* models (THP-1 cell line, murine BMDM and human monocytic cells) (38). Another group used quenched activity-based probe BMV109 for monitoring pan-cathepsin activity in a mouse model of breast cancer metastasis to bone. Even though cathepsins B, L, S, and X were abundant in MDSC, blocking their activity with small-molecule inhibitors did not elevate the suppressive effect during an *in vitro* T lymphocyte proliferation assay. The impact of inhibitors on MDSC expansion *in vivo* was, however, not tested (47). Among several tumor-promoting roles, MDSC can infiltrate bone marrow and bolster bone metastasis by differentiation into functional osteoclasts (208). For such differentiation to occur, the activities of cathepsins B, L, and X need to be down regulated. Osteoclast fusion was accelerated in the presence of CA074 and JPM-OEt, suggesting that cathepsin inhibitors could potentiate bone degradation and metastasis (47).

Cathepsin K is a key osteoclast collagenase involved in bone resorption. More, in an SCID-hu mouse bone tumor model, cathepsin K was found to interfere with macrophage-regulated inflammatory processes. Cathepsin K contributed to tumor macrophage recruitment via increased levels of chemokine CCL2. Additionally it promoted overexpression of cathepsin B and COX2, two other important drivers of tumor progression (42). COX2 is often amplified in cancer, leading to increased synthesis of eicosanoid PGE2, which is important for functional differentiation and immunosuppressive properties of TAM and MDSC (209, 210). Cathepsins can enhance PGE2 production as they are involved in post-translational COX2 maturation and catalytic regulation. Broad-spectrum cathepsin inhibitors E64d and ALLn can block COX2 maturation, thus resulting in diminished PGE2 formation (43). On the other hand, the expression of chemokine CCL2, which is strongly associated with recruitment of MDSC and TAM, depends specifically on cathepsin S activity. MHC II chaperone invariant chain (also CD74) has, besides its well-documented role in antigen presentation, additional functions in promoting gene transcription. Cathepsin S cleavage of CD74 in endosomes results in release of CD74 intracellular domain, its nuclear translocation and activation of transcription factor NF-κB, finally resulting in CCL2 transcription (44).

### Cathepsins and Chemotherapeutic Modulation of Myeloid Cells

Cysteine cathepsins can even be implicated in resistance to chemotherapeutic drugs 5-fluorouracil and gemcitabine that selectively kill MDSC. The off-target effect of both agents is lysosomal membrane permeabilization, followed by cathepsin B release in the cytosol and NLRP3 inflammasome activation. Consecutive production of cytokine IL-1β stimulates CD4+ T lymphocytes to produce IL-17 which in turn accelerates tumor growth (48). LCL521, a lysomotropic inhibitor of acid ceramidase, is another agent found to induce lysosomal cell death of MDSC by activating cathepsin B (211). Also, Shree at al. demonstrated that the common chemotherapeutic paclitaxel, markedly increased TAM infiltration to the tumor site, thereby contributing to local increase in cathepsin activity. As shown in an *in vitro* co-culture system, macrophage-secreted peptidases protect against paclitaxel, etoposide and doxorubicin induced tumor cell death. The effect was attributable to cathepsins S and B but not to cathepsins C and L. Importantly, addition of pan-cathepsin inhibitor JPM improved the response to chemotherapy *in vivo* (45).

Proteomic analysis of MDSC from metastatic tumors revealed a decrease of neutrophilic granule protein, NGP, compared to that of non-metastatic counterparts. NGP was originally discovered in immature bone marrow cells and in promyelocytes and is structurally similar to type II cystatins. NGP was shown to be capable of inhibiting cathepsin B and *in vivo* reduced tumor vascularization, tumor growth and metastasis (49). Doubtless, cysteine cathepsins are tightly involved in control of the biochemical processes that shape MDSC/TAM cell survival, differentiation, and functional status and the lack of control over cathepsin activity appears to facilitate differentiation of immature myeloid cells into more potent immunosuppressive effectors.

## Cysteine Cathepsins in Lymphoid Cells—Regulating Immune Cell Cytotoxicity

The role of cysteine cathepsins has also been studied extensively in the lymphoid immune cell lineage. Cathepsins C, H, and L, in particular, have been implicated in the ability of CTL and NK cells to kill target cells via the perforin/granzyme pathway. In T lymphocytes, cysteine peptidase cathepsin X associates with integrin LFA-1, which is necessary for T lymphocyte migration, activation and formation of stable immunological synapse. In the following chapter the contribution of cysteine cathepsins to both processes will be discussed.

### LFA-1 and Immunological Synapse

Interactions between LFA-1 and its ligands, intercellular adhesion molecules, are at first necessary for establishing low affinity exploratory contacts between target and effector cells. Secondly, on target cell recognition, activated LFA-1 molecules organize in a specific manner to form a peripheral ring of the immunological synapse. The adhesive ring encompasses spatially segregated signaling domain, termed central supramolecular activation cluster, that consists of TCR ligated to cognate MHC molecule on APC (212, 213). However, compelling evidence demonstrates that the role of LFA-1 goes beyond that of simple adhesion. Depending on the type of interacting cell, LFA-1 receptor was shown to modulate gene expression and to direct T lymphocyte development, differentiation and effector function (214, 215). Tumor specific CTL, deficient in LFA-1 molecule, fail to reject immunogenic tumors (216). LFA-1 blockade affects the stability of the immunological synapse, impairs microtubule-organizing center polarization and actin redistribution at the site of contact between APC and CTL (217). LFA-1 engagement results in a distinct signature of activated kinases and phosphoproteins, indicating that LFA-1 synergistically contributes to modulation of T lymphocyte functions. Indeed, it enhances T lymphocyte proliferation and IL-2 transcription and favors Th1 differentiation of CD4+ T lymphocytes (218, 219). LFA-1 co-stimulation also reduces the amount of antigen needed to trigger the T lymphocyte activation (220) and is important for Treg development and function. Mice without LFA-1 are susceptible to autoimmunity due to abrogated development of functional Treg (221). On the other hand, LFA-1-ICAM-1 interactions promote T lymphocyte refractoriness to cytokine TGF-β, a known inducer of Treg (222).

Talin 1 regulates LFA-1 function at the very onset of immunological synapse formation by coupling LFA-1 to the actin cytoskeleton and increasing LFA-1 affinity and clustering (56). Moreover, talin 1 ablation in mice resulted in similar impairment of Treg frequencies and suppressive capacity, as seen in LFA-1 deficient mice, additionally supporting the requirement for talin 1 in LFA-1 integrin signal transduction (223). It is not yet clear how talin 1 is recruited to LFA-1. Its association with LFA-1 could be promoted by conformational changes induced in talin 1 by phosphatidylinositol 3,4-bisphosphonate or by cleavage by calpain 2 that leads to increased affinity of talin 1 for integrin β subunit tail (56). Cathepsin X could be another potential candidate, since it introduces conformational changes to the talin-binding site that strengthen the interaction of talin 1 with integrin LFA-1 (224). Profilin 1, another protein that controls actin dynamics, has been shown to downregulate CTL migration and lytic granule release, acting as a negative regulator of CTL cytotoxicity (57). Remarkably, profilin 1 was also validated as a cathepsin X target. Cathepsin X mediated profilin 1 cleavage is important for ligand binding and for actin polymerization (55, 225). These studies suggest that cathepsin X could be involved in modulating integrin signaling during immunological synapse formation.

### Immune Cell Cytotoxicity

Effective antitumor immune response is based on the ability of NK cells and CTL to recognize and destroy cancer cells. Innate NK cells exist in a pre-activated state while adaptive CTL need to be primed by APC. However, both employ the same killing mechanisms, executed either through death receptor pathway or cytotoxic granule release (226). Cytotoxic granules are lysosome-like secretory vesicles (227) containing pro-forms of perforin and several granzymes (granzymes A, B, H, M, and K in humans), of which granzymes A and B are the most abundant (228). Perforin is a calcium dependent pore forming protein that needs to be truncated at the C terminus by 20 amino acids to liberate its C2 domain. Perforin binding to the cell membrane is needed for the entry of granzymes into target cells, where they can trigger apoptosis (229). Granzymes are serine proteases, stored in cytotoxic granules as inactive precursors that require N-terminal dipeptide removal for activation (230). Given the acidic milieu in cytotoxic granules and the presence of lysosomal cathepsins, these were studied as the most likely pro-perforin and pro-granzyme convertases. At first perimembrane cathepsin B was believed to protect cytotoxic cells from self-destruction by digesting and inactivating perforin molecules (231). However, later experiments in a more relevant *in vivo* model rejected these claims, since CTL from mice deficient in cathepsin B survived as well as that from wild-type mice. Moreover, *in vitro*, cathepsin B processed purified perforin very poorly (232). Next, cathepsin C was also found to be dispensable for perforin processing in human NK cells (59) and, finally, cathepsin L was identified as cysteine peptidase with the ability to generate C-terminally truncated perforin. Cathepsin L inhibition could diminish perforin activation and reduce killing capacity in human NK cell lines and primary mouse CTL. Nevertheless, cathepsin L deficiency *in vivo* had no impact on cytotoxicity, despite reduction in the amount of active perforin (58). Other, still unknown convertases can compensate for the loss, ensuring that the host immune response is not compromised.

Similar observations were made for the role of cysteine cathepsins in granzyme processing. Cytotoxic cells isolated from cathepsin C^−/−^ mice were reported to be defective in inducing target cell apoptosis (60). Albeit cathepsin C has an essential role in the *in vivo* activation of granzymes A and B, residual granzyme B activity is sufficient to combat viral infection in cathepsin C null mice (233). Reduced expression of β_2_ integrin receptor CD11c on DC and CD11c or CD11b on CD8+ T lymphocytes was observed in mice with a genetic deficiency of cathepsin C. Since β_2_ integrins are important for several aspects of immune cell function, cathepsin C deficiency may not have fatal but still significant consequences (234). Not only in mouse, but also in humans with Papillon-Lefèvre syndrome, marked by severe reduction in cathepsin C activity, cytotoxic cells display adequate killing ability (235). At least for granzyme B activation, cathepsin H, another aminopeptidase, has been shown to possess convertase activity. However, even in mice lacking both cathepsin C and H, the granzyme B activity was not completely diminished, suggesting the involvement of other convertases (61). Cathepsin C is also needed for processing serine protease cathepsin G in neutrophils, and Papillon-lefevre syndrome patients may suffer from the consequences of neutrophil dysfunction (235).

Endogenous cysteine cathepsin inhibitor, cystatin F, is a major regulator of cathepsin C, H, and L activities in cytotoxic immune cells (84, 85, 236). Notably, when a state of functional anergy was induced, in either NK cells or CTL, cystatin F levels increased, while levels of cathepsins C, H, L, and granzyme B levels and activities declined (84, 236). Even though cystatin F is, to a greater extent, targeted to endosomes, part of it is secreted as an inactive dimer. Bystander cells are able to internalize dimeric cystatin F and convert it to the active monomer (85, 237). The regulatory ability of *in trans* cystatin F could be exploited by cancer cells that, together with cancer stem cells and monocytes, overproduce and secrete cystatin F, in order to lower the antitumor immune cell cytotoxicity (85). Not only the activity, but also protein levels of cathepsin C could be modulated in cytotoxic cells in response to different activating stimuli. Sanchez-Martinez et al. have shown that cathepsin C expression is negatively regulated by micro RNA, miR-23a, which needs to be downregulated for successful granzyme B processing. Interestingly, a known immunomodulatory agent, all-trans retinoic acid, inhibits NF-κB activation and hence promotes increase in miR-23a levels (238). Since all-trans retinoic acid has also been used in clinical trials to deplete MDSC by differentiating them to DC and macrophages (239), its long term effects on cytotoxic cell fitness in cancer patients deserve further evaluation.

Recently, a novel mechanism of regulating cytotoxicity was described for cathepsin L. After stimulating the T lymphocyte receptor, cathepsin L cleaves complement component C3 into C3a and C3b fragments that activate their corresponding receptors (C3aR and CD46) in an autocrine manner. Engagement of C3aR and CD46 in CD4 + T lymphocytes improves their survival and favors Th1 differentiation (62) while, in CD8+ T lymphocytes, signaling via CD46 is needed for optimal cytotoxic activity (63). It was shown for CD4+ lymphocytes that CD46 co-stimulation induces AEP expression which acts as an upstream regulator of cathepsin L activity (240).

Although not directly implicated in tumor cell killing, CD4+ lymphocytes importantly determine the course of anti-tumor immune response through the array of cytokines they secrete. For example specific subset of T helper lymphocytes, Th17 cells, plays dynamic role in cancer-related inflammation, which is sometimes detrimental or beneficial, depending on the context and cancer type (241). It has been shown recently that cathepsin S and L are both involved in Th17 cell differentiation. After exposure to LPS from *Porphyromonas gingivalis* cathepsin S could activate PAR2 receptor on DC cells, stimulating them to produce IL-6 that drove splenic Th17 expansion (242). In contrast to cathepsin S, which generates Th17 cells via DC-dependent mechanism, cathepsin L is a CD4+ cell intrinsic promoter of Th17 development. It has been shown that mouse CD4+ cells more readily differentiate to Th17 cell type when lacking serpin B1, an endogenous cathepsin L inhibitor. Differentiation could be blocked by addition of specific exogenous cathepsin L inhibitor (243). Cathepsins are also important for fine-tunning immunobiological activities of Treg. Cathepsin S inhibition enhances immunosuppressive activity of Treg under normal conditions, possibly by reducing B cell, CD4+ and CD8+ T lymphocyte proliferation. However, under influence of tumor cells cathepsin S inhibited Treg rather stimulate anti-tumor immunity by promoting CD8+ lymphocyte proliferation and survival. (244) Similar observations have been made for Treg from mice lacking cathepsin K, which turned out to be more potent suppressors of effector T lymphocytes. Of note, it was not tested whether their function changes in the presence of tumor cells as well (245). Apart from these studies, CD4+ cell cathepsins have received little attention in comparison to other immune cell types.

## Conclusions

The emerging trend in cancer therapy is to enhance cytotoxic immune response by activating cytotoxic cells in order to achieve effective killing of malignant cells and to reprogram myeloid cells toward the anti-tumor phenotype. As important constituents of the tumor microenvironment, immune cells support either rejection or progression of the tumor. Overexpression and increased activity of cysteine cathepsins in myeloid cells most probably fosters generation of tumor promoting immunosuppressive cells such as TAM and MDSC. In contrast, repression of cathepsins L, C, and H in CTL and NK cells results in attenuated tumor cell lysis. Understanding the differential regulation of cysteine cathepsins' expression and activity in normal and pathologically activated myeloid cells is of vital importance. For decades cysteine cathepsins have been recognized as viable therapeutic targets in cancer treatment. However, given the relevance of their immunoregulatory roles, inhibiting cysteine cathepsins could have unexpected effects on the anti-cancer immune response. It could result for example, in activation of alternative transcriptional factors, like STAT3, signaling *de novo* synthesis of lysosomal enzymes (246). Alternatively, it could induce expression of related peptidases through a positive feedback loop, as shown by genetic deletion of cathepsin B in tumor bearing mice that can be counteracted by a compensatory increase in cathepsin X expression (247). In addition, it has been shown that inhibiting cathepsin S in Treg may provide contrasting effects on immune system, depending on whether they were exposed to tumor microenvironment or grown under normal conditions (244). The application of specific inhibitors of cysteine cathepsins in cancer *in vivo* models may alter the immune system functions, an issue that needs to be better considered in further studies.

## Author Contributions

TJ designed the concept and drafted manuscript in consultation with AP and JK. AP, AJ, and JK completed and revised the content. TJ prepared the figure.

### Conflict of Interest Statement

The authors declare that the research was conducted in the absence of any commercial or financial relationships that could be construed as a potential conflict of interest. The reviewer MP declared a past co-authorship with several of the authors AP and AJ to the handling editor.

## Abbreviations

AEP: Asparaginyl endopeptidase
legumain
APC: Antigen presenting cell/s
Arg1: Arginase 1
BMDM: Bone marrowderived macrophages
COX2: Cyclooxygenase 2
CTL: Cytotoxic T lymphocytes (CD8+ T lymphocytes)
DC: Dendritic cell/s
ECM: Extracellular matrix
GILT: Gamma-interferon-inducible lysosomal thiol reductase
ICAM: Intercellular adhesion molecules
IDO, Indoleamine-2: 3-dioxygenase
Ii: Invariant chain
iNOS: Inducible nitric oxide synthase
LFA-1: Lymphocyte-function-associated antigen-1
LPS: Lipopolysaccharide
MHC: Major histocompatibility complex
MDSC: Myeloid-derived suppressor cells
NK: Natural killer
NOX2: NADPH oxidase 2
PGE2: Prostaglandin E2
ROS: Reactive oxygen species
STAT: Signal transducer and activator of transcription
TAM: Tumor-associated macrophages
TLR: Toll-like receptor/s
Treg: Regulatory T lymphocytes.
